# *Brucella* β 1,2 Cyclic Glucan Is an Activator of Human and Mouse Dendritic Cells

**DOI:** 10.1371/journal.ppat.1002983

**Published:** 2012-11-15

**Authors:** Anna Martirosyan, Camino Pérez-Gutierrez, Romain Banchereau, Hélène Dutartre, Patrick Lecine, Melissa Dullaers, Marielle Mello, Suzana Pinto Salcedo, Alexandre Muller, Lee Leserman, Yves Levy, Gerard Zurawski, Sandy Zurawski, Edgardo Moreno, Ignacio Moriyón, Eynav Klechevsky, Jacques Banchereau, SangKon Oh, Jean-Pierre Gorvel

**Affiliations:** 1 Centre d'Immunologie de Marseille-Luminy (CIML), Aix-Marseille University, UM2, Marseille, France; 2 Institut National de la Santé et de la Recherche Médicale (INSERM), U1104, Marseille, France; 3 Centre National de la Recherche Scientifique (CNRS), UMR7280, Marseille, France; 4 Fundación Investigación Sanitaria Illes Balears-CSIC, Bunyola, Spain; 5 Baylor Institute for Immunology Research and INSERM U899, Dallas, Texas, United States of America; 6 Laboratory of Immunoregulation and Mucosal Immunity, Department of Pneumology, Gent, Belgium; 7 IINSERM U955 Equipe 16, Institut Mondor de Recherche Biomédicale, Créteil, France; 8 Université Paris-Est, Faculté de Médecine, UMR-S 955 Créteil, France; 9 Assistance Publique-Hôpitaux de Paris (AP-HP), Groupe Henri-Mondor Albert-Chenevier, 9 service d'immunologie clinique, Créteil, France; 10 Programa de Investigación en Enfermedades Tropicales, Escuela de Medicina Veterinaria, Universidad Nacional, Heredia, Costa Rica; 11 Centro de Investigación en Enfermedades Tropicales, Universidad de Costa Rica, San José, Costa Rica; 12 Instituto Clodomiro Picado, Universidad de Costa Rica, San José, Costa Rica; 13 Institute for Tropical Health and Departamento de Microbiología y Parasitología, Universidad de Navarra, Pamplona, Spain; 14 Department of Pathology and Immunology, Washington University School of Medicine, St. Louis, Missouri, United States of America; 15 Pharma Research and Early Development, Hoffmann-La Roche, Nutley, New Jersey, United States of America; University of California, Davis, United States of America

## Abstract

Bacterial cyclic glucans are glucose polymers that concentrate within the periplasm of alpha-proteobacteria. These molecules are necessary to maintain the homeostasis of the cell envelope by contributing to the osmolarity of Gram negative bacteria. Here, we demonstrate that *Brucella* β 1,2 cyclic glucans are potent activators of human and mouse dendritic cells. Dendritic cells activation by *Brucella* β 1,2 cyclic glucans requires TLR4, MyD88 and TRIF, but not CD14. The *Brucella* cyclic glucans showed neither toxicity nor immunogenicity compared to LPS and triggered antigen-specific CD8^+^ T cell responses *in vivo*. These cyclic glucans also enhanced antigen-specific CD4^+^ and CD8^+^ T cell responses including cross-presentation by different human DC subsets. *Brucella* β 1,2 cyclic glucans increased the memory CD4^+^ T cell responses of blood mononuclear cells exposed to recombinant fusion proteins composed of anti-CD40 antibody and antigens from both hepatitis C virus and *Mycobacterium tuberculosis*. Thus cyclic glucans represent a new class of adjuvants, which might contribute to the development of effective antimicrobial therapies.

## Author Summary

Vaccination is one of the key strategies to fight against infectious diseases though numerous diseases remain without appropriate vaccines. The challenge is to generate potent vaccines capable of inducing long-lasting immunity in humans. Successful vaccines include adjuvants that enhance and appropriately skew the immune response to given antigens. The development of new adjuvants for human vaccines has become an expanding field of research. Here we show that bacterial cyclic β-glucans can be used to enhance cellular immunity by activation of dendritic cells, from both mice and humans. In particular, Cyclic-β glucans enhance the *in vitro* memory CD4+ T cell responses of patients suffering from hepatitis C and tuberculosis. Thus cyclic-β glucans are new adjuvants, which might be used in vaccines.

## Introduction

Cyclic glucans are intrinsic components of the envelopes of Gram negative bacteria such as *Agrobacterium*, *Rhizobium*, *Ralstonia solanacearum*, *Xanthomonas campestris*, *Rhodobacter sphaeroides* and *Brucella spp.* [1]. Brucellae are intracellular pathogens of mammals, including humans [2]. The pathogenesis of brucellosis is linked to the ability of *Brucella* to survive and replicate inside host cells [3] through the expression of several effector molecules [2]. In particular, the periplasmic cyclic glucan is required for *B. abortus* intracellular trafficking [4]–[6] through the recruitment of the raft protein flotilin-1 at the site of the *Brucella*-containing vacuole [6]. This polysaccharide is built of a cyclic backbone of 17 to 25 glucose units in β-1,2 linkages (CβG). CβG are abundant as they represent 1–5% of the bacteria dry weight. If the CβG content of a single bacterium is released inside a *Brucella*-containing vacuole, its concentration in the vacuole can reach 10 mM. The release of CβG in µM concentration upon bacterial killing through immune mechanisms [6] might alter the host immune responses.

While linear (1, 3) β-glucans have been shown to elicit anti-tumor [7], [8] and anti-infective [9]–[12] responses [13], [14], we do not know whether CβG displays immunomodulatory functions. In this study, we have analyzed the effects of CβG on mouse and human dendritic cells (DC) and its consequences on immune responses.

The discovery of vaccination is one of most important medical discoveries in the history and a turning point in the war between microbes and humans [15], [16]. The goal of vaccination is to induce long-lasting protective immunity from infection and prevent infectious diseases. Vaccines operate through the activation of antigen-presenting cells such as DC that eventually stimulate antigen-specific T and B lymphocytes. Unlike attenuated live vaccines, killed whole organisms or subunit vaccines generally require the addition of adjuvants to be effective. Adjuvants promote and enhance immune responses to vaccine components [15], [16]. It is now clear that adjuvants activate DC [15]. Adjuvants derived from microorganisms stimulate DC directly, leading to the up-regulation of cytokines, MHC class II, and co-stimulatory molecules and to their migration to the T cell area of lymph nodes. These pathogen-associated molecular patterns (PAMP) activate pattern-recognition receptors (PRR), which act as microbial sensors and are expressed by DC and other leukocytes [13], [14]. Most of PAMP used as vaccine adjuvants, like CpG oligonucleotides and monophosphoryl lipid A (MPLA), are agonists of Toll-like receptors (TLR) [17], [18]. For infectious as well as for noninfectious diseases, TLR activation have been used in both established and experimental vaccines [17], [18].

Bacterial components are often potent immune activators trough commonly associated with toxicity, for example, bacterial DNA with immunostimulatory CpG motifs that bind TLR-9 are potent cellular adjuvants. Overall, several hundred natural and synthetic compounds have been identified to have adjuvant activity such as microbial products, mineral salts, emulsions, microparticles, and liposomes. Although many are more potent than alum, the almost universal human vaccine adjuvant, toxicity is the limiting step for their use in humans. Consequently there is a major unmet need for safer and more effective adjuvants suitable for human use [15]–[17].

We show here that *Brucella* CβG is neither toxic nor immunogenic when compared to LPS. It is a potent activator of DC thereby triggering antigen-specific CD8^+^ T cell responses *in vivo. Brucella* CβG enhance antigen-specific CD4^+^ and CD8^+^ T cell responses including cross-presentation by different human DC subsets.

## Results

### *Brucella cgs-* mutants are poor inducers of DC maturation

Wild type *B. abortus* triggers limited activation of mouse bone marrow-derived dendritic cells (BMDC) [19]. Infection of BMDC with *B. abortus cgs*- (cyclic glucan synthase) mutant failed to activate BMDC as measured by the production of TNF-α and IL-12 (Figure S1A and S1B). Likewise these infected DC displayed a low expression of immune co-stimulatory molecules such as CD80, CD83 and CD86 (not shown). This pilot findings led us hypothesize that CβG indeed acts as a DC activation molecule. To this end we had to ensure that the CβG preparations would not be contaminated by the potent DC activators LPS and lipid A. Whereas CβG are highly soluble in water, *B. abortus* and *B. melitensis* LPS partition in the phenol phase of the classical Westphal hot water-phenol extraction procedure. Thus, this extraction method was applied twice to a CβG water extract previously digested with nucleases and proteinase K. The identity of the CβG was established by several methods, including ^13^C-NMR, and the absence of LPS tested by both conventional analytical methods (SDS-PAGE, inability to elicit anti-LPS antibodies, and Kdo analysis). MALDI-TOF analysis further showed both the spectra expected from *Brucella* CβG and the absence of molecular species signalling like *Brucella* lipid A (Figure S2A and S2B).

### Cyclic glucan activate murine DC

**Figure 1:**
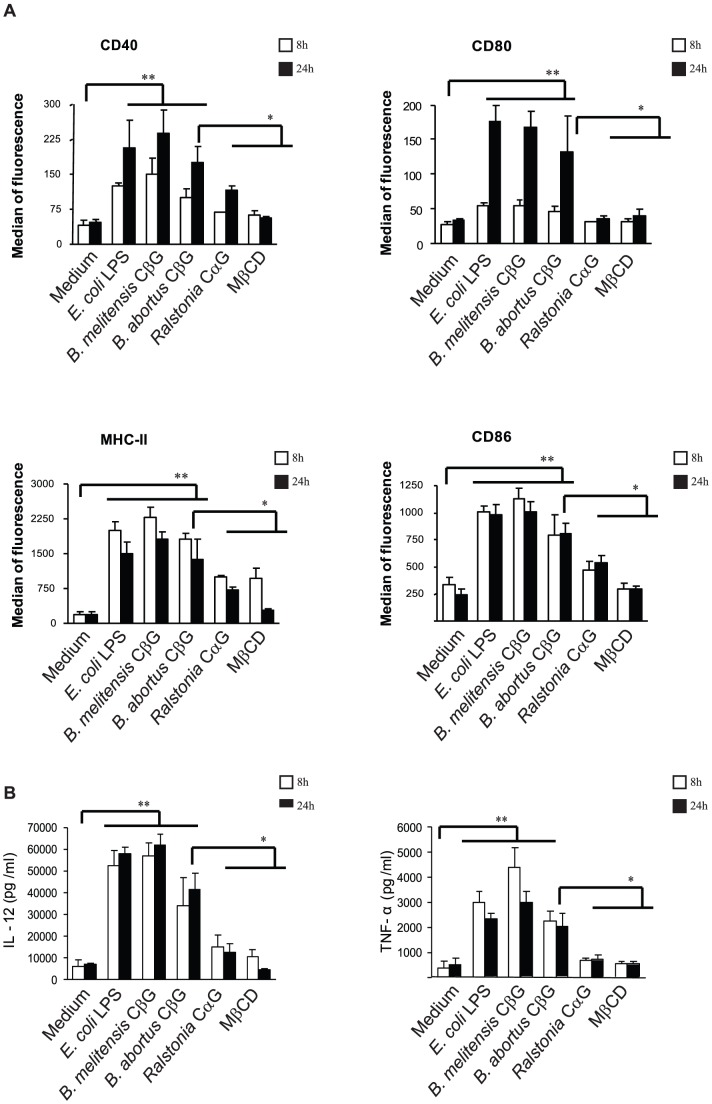
Induction of mouse BMDC maturation depends on the structure of the cyclic glucan. Mouse BMDC were stimulated for 8 h (in white) and 24 h (in black) with medium, *E. coli* LPS, *B. melitensis* CβG, *B. abortus* CβG, *Ralstonia* CαG or MβCD at equivalent molarity (0.25 µM). Surface levels of MHC-II, CD80, CD40 and CD86 were measured by flow cytometry (**A**). IL-12 and TNF-α secretion levels in culture supernatant were determined by ELISA (**B**). Data are representative of at least three independent experiments.* p<0.05, ** p<0.01.

Mouse BMDC were incubated with synthetic methyl-β-cyclodextrin (MβCD) and cyclic glucans purified from *Brucella melitensi*s, *Brucella abortus and Ralstonia solanacearum*. Brucella CβG consists of a cyclic backbone of 17–25 glucose residues linked in β-(1→2) associated with O-succinyl modifications [20], [21]. Ralstonia cyclic α-glucan (CαG) is composed of 12 glucoses linked in eleven β-(1→2) plus one α-(1→6) linkages [20]. MβCD consists of 7 O-methyl substituted glucoses in β-(1→4) linkages. Both *B. melitensis* and *B. abortus* CβG induced DC to express levels of CD80, CD86, CD40 and MHC II molecules, comparable to those elicited by *E. coli* LPS (Figure 1A). The two *Brucella* CβG induced the secretion of high levels of pro-inflammatory cytokines such as TNF-α and IL-12 (Figures 1B). The induction was dose-dependent (Figure S3A and S3B). These findings contrast with the poor DC-activating ability of *Brucella* LPS [2]. When compared to *Brucella abortus* CβG, the synthetic MβCD did not stimulate DC (**p<0.01) and the *Ralstonia* CαG hardly induced (**p<0.01) the production of TNF-α and IL-12 (Figure 1B). Thus *Brucella melitensis* CβG is a potent activator of mouse DC.

### DC activation by CβG requires TLR4, MyD88 and TRIF, but not CD14

**Figure 2:**
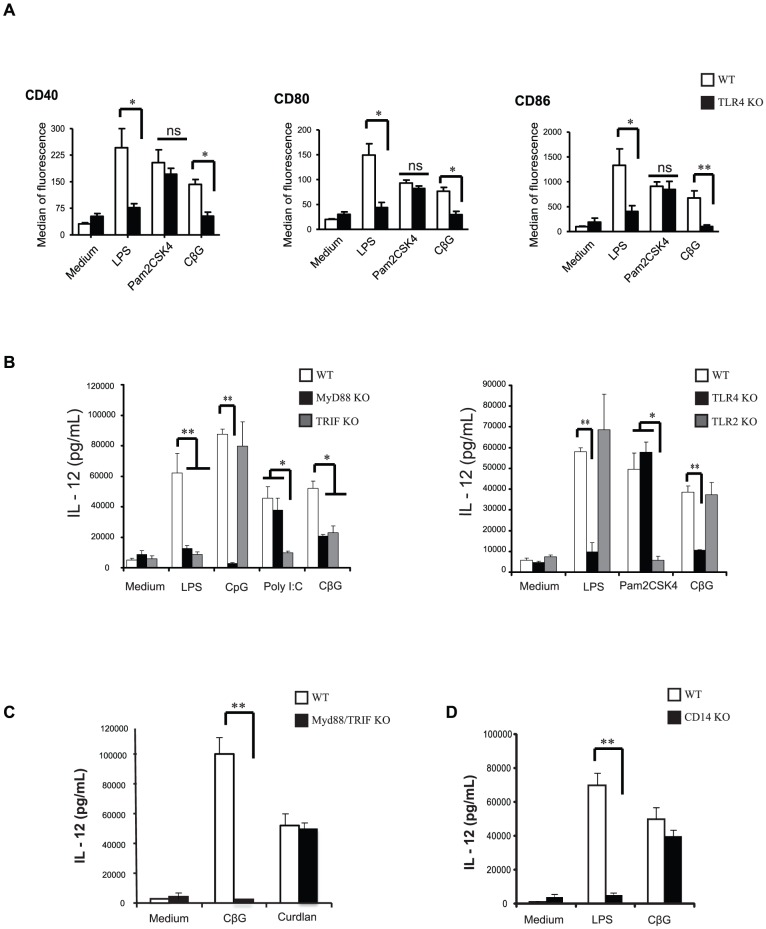
CβG-induced BMDC maturation is TLR4-dependent. (**A**) BMDC from wild type (white bars) and TLR4^−/−^ (black bars) mice were stimulated either with *E. coli* LPS (0.25 µM), *B. melitensis* CβG (0.25 µM) or Pam2CSK4 (10 ng/ml). Cell maturation was assessed by flow cytometry for surface co-stimulatory molecule expression (CD40, CD80 and CD83). Data are representative of at least three independent experiments. * p<0.05, ** p<0.01, ns: not significant. (**B**) Left panel: BMDC from wild type (white bars), MyD88^−/−^ (black bars) and TRIF^−/−^ (grey bars) mice were treated with either *E. coli* LPS (0.25 µM), *B. melitensis* CβG (0.25 µM), CpG (1 µM) or Poly I:C (50 µg/ml). IL-12 secretion level was measured by ELISA. Right panel: BMDC from wild type (white bars), TLR4^−/−^ (black bars) and TLR2^−/−^ (grey bars) mice were activated by *E. coli* LPS (0.25 µM), *B. melitensis* CβG (0.25 µM) or Pam2CSK4 (10 ng/ml). IL-12 secretion level was determined by ELISA. Data are representative of at least three independent experiments. * p<0.05, ** p<0.01. (**C**) IL-12 secretion level was analyzed in BMDC from wild type (white bars) and MyD88/TRIF^−/−^ (black bars) mice stimulated by *B. melitensis* CβG (0.25 µM) or curdlan (100 µg/ml). Data are representative of at least three independent experiments.** p<0.01. (**D**) IL-12 secretion level was analyzed in BMDC from wild type (white bars) and CD14^−/−^ (black bars) mice were stimulated by *E. coli* LPS (0.25 µM) or *B. melitensis* CβG (0.25 µM). Data are representative of at least three independent experiments. ** p<0.01.

We then asked whether CβG, like LPS would activate DC through TLR4, MyD88 and TRIF pathways. Thus, BMDC were derived from TLR4^−/−^, TLR2^−/−^, MyD88^−/−^, TRIF^−/−^, TRIF/MyD88^−/−^ and CD14^−/−^ mice. These DC were activated with either CβG or different TLR agonists such as CpG (TLR9 agonist), Pam2CSK4 (TLR2/6 agonist), curdlan (linear β-1,3 glucan from *Alcaligenes faecalis* agonist of Dectin-1 [22], [23]) and *E. coli* LPS (TLR4/MD2/CD14 agonist). Neither *E. coli* LPS nor *B. melitensis* CβG were able to induce the expression of co-stimulatory molecules (Figure 2A) and secretion of IL-12 (Figures 2B, 2C) by BMDC from TLR4^−/−^, Myd88^−/−^, Myd88/TRIF^−/−^ and TRIF^−/−^ mice. In addition, transfection of HEK 293T cells with vectors coding for TLR4/MD2, TLR2, TLR3 and TLR9 showed that CβG effect is dependent on TLR4/MD2 (not shown). Moreover, CβG-treated human blood plasmacytoid DC (pDC) known to be devoid of surface TLR4 expression [24]–[26] were not activated by any of these agents (data not shown). These results show that DC activation by CβG is TLR4-dependent and that DC activation is dependent on both MyD88 and TRIF adaptors (Figure 2B, left panel).

We then compared DC activation induced by *B. melitensis* CβG to that induced by linear ß1-3 glucans (curdlan), which bind to Dectin-1 and activate DC in a MyD88/TRIF-independent manner [22], [23]. First, several monoclonal antibodies specific for Dectin-1 that can inhibit curdlan-mediated activation failed to inhibit CβG-mediated DC activation (not shown). While double MyD88/TRIF^−/−^ BMDC did not respond to CβG (**p<0.01), they secreted high levels of IL-12 in response to curdlan (Figure 2C). These results indicate that CβG and curdlan use different signalling pathways and that Dectin-1 is not the receptor for CβG.

LPS recognition involves the LPS-binding protein (LBP), the TLR4/MD2 complex and CD14 [27]. Accordingly, CD14^−/−^ DC did not secrete IL-12 upon exposure to *E. coli* LPS (Figure 2D). CD14^−/−^ DC also failed to upregulate co-stimulatory molecules and MHC-II in response to LPS (not shown). Strikingly, CβG was able to stimulate CD14^−/−^ DC to secrete IL-12 (Figure 2D).

Altogether, these data show that CβG signalling is dependent on TLR4 but does not use CD14 as a co-receptor.

### *Brucella* CβG is neither toxic nor immunogenic

LPS displays a toxicity that precludes its use as a vaccine adjuvant. Previous studies in cell cultures revealed that CβG, when compared to MβCD was not cytotoxic even at very high concentrations [6]. In addition, the Limulus Ameobocyte Lysate (LAL) test showed that CβG preparations did not contain significant endotoxin levels. To assess CβG toxicity in mice LD50 (Lethal dose 50%) was determined by injecting increasing amounts with death being recorded at 12 h, 24 h, 48 h, 72 h post-injection. Results showed that more than 500 µg of *Brucella* CβG were required to kill 50% of mice, when compared to 65 µg of *E. coli* LPS.

To determine the immunogenicity of CβG, Balb/C mice were injected with by PBS, *E. coli* LPS, *B. melitensis* LPS or *B. melitensis* CβG. Primary and secondary antibody responses were analyzed and total immuoglobulin levels quantified by ELISA. Unlike *E. coli* LPS or *B. melitensis* LPS, *B. melitensis* CβG did not induce the generation of specific antibodies (Figure S4A). We also analyzed CβG-mediated ability to induce pro-inflammatory cytokines in the sera of immunized mice by Cytokine Bead Array (CBA) assay (Figure S4B). C57Bl/6 mice were injected with PBS, CβG, monophosphoryl-lipid A (MPLA), LPS or Poly I/C. After 6 h, 24 h and 72 h of immunization, mice were bled and cytokine levels were measured. At 6 h post-immunization CβG, MPLA and PolyI/C did not induce pro-inflammatory cytokine secretion in contrast to *E. coli* LPS (Figure S4B). Altogether, these data show that *Brucella* CβG is neither toxic nor immunogenic in mice.

### *Brucella* CβG increases antigen-specific CD8^+^ T cell responses *in vivo*

**Figure 3:**
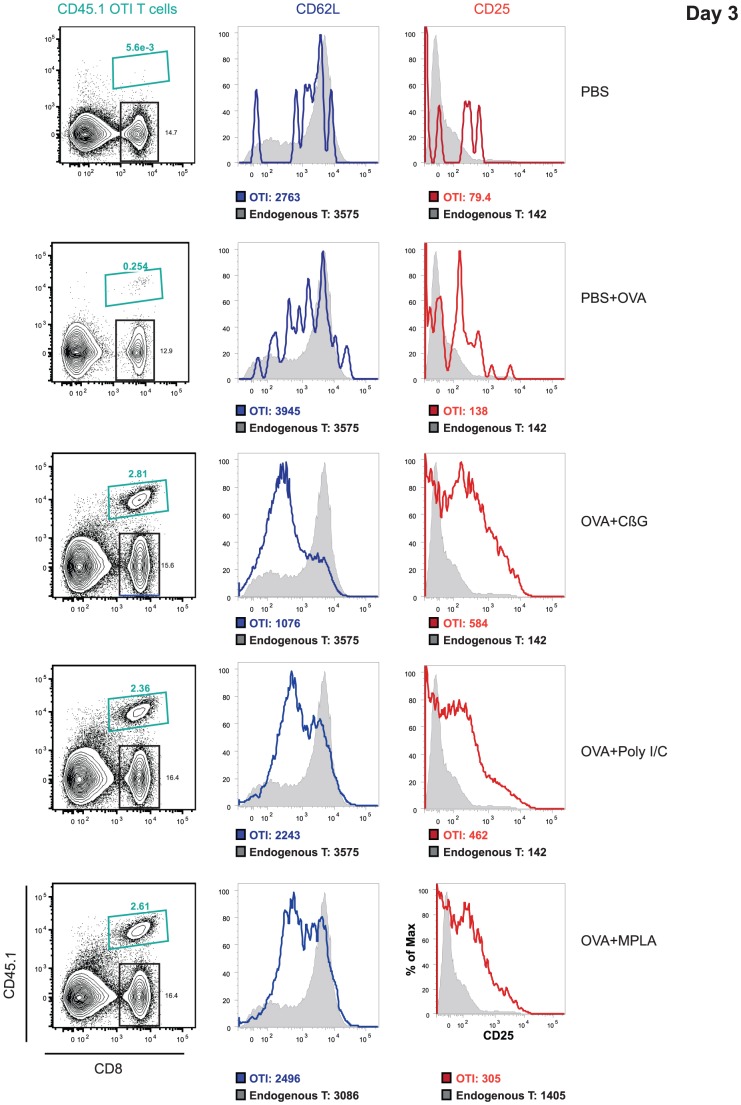
*B. melitensis* CβG induces cellular and responses *in vivo*. The capacity of *Brucella* CβG to induce OVA-specific CD8+ T cell responses was evaluated. CD8^+^ Ly5.2 CFSE^+^ T cells were transferred intravenously (i.v.) into naive congenic C57Bl/6 Ly5.1 recipient mice (n = 10). At 24 h, recipient mice were immunized subcutaneously (s.c.) either with 30 µg OVA in endotoxin free PBS or 30 µg OVA mixed with 200 µg of CβG or 30 µg OVA mixed with 50 µg poly I:C (Sigma) or 30 µg OVA mixed with 20 µg MPLA (InvivoGen). At day 3 post-immunization, the OVA-specific OT-I T cell activation in the draining popliteal lymph nodes of immunized mice was investigated by analyzing the up-regulation of CD25 and the down-regulation of CD62L by flow cytometry. The median fluorescence for each marker is indicated under histograms. Endogenous CD8^+^ T cell population (in grey). Data are representative of one experiment among three different experiments.

Given its ability to activate DC and its low toxicity profile we wondered whether administration of CβG to mice would enhance antigen-driven cellular immune responses *in vivo*. We used transgenic mice (OT-I Rag^−/+^) that express a CD8^+^ T cell population specific for ovalbumin (OVA) as well as a congenic Ly5.1/Ly5.2 mouse model with 2 allelic forms of CD45. We transferred CD8^+^ Ly5.2 CFSE^+^ OT-I T cells into C57Bl/6 Ly5.1 mice, and immunized them subcutaneously with either OVA alone, or a mix of OVA with CβG or OVA with Poly I:C or OVA with MPLA [15]. At day 3 post-immunization, OVA-specific OT-I T cell proliferation of all immunized mice was detected in the draining lymph nodes (Figure 3). OVA-specific T cells from mice immunized with Poly I:C, MPLA and CβG showed an up-regulation of CD25 and a down-regulation of CD62L, which correlated with T cell migration from lymph nodes to the sites of infection. OVA-specific T cells from mice treated with CβG showed a stronger down-regulation of CD62L expression than those treated with Poly I:C or MPLA (Figure 3).

At day 6 post-immunization (Figure S5), OVA-specific T cells from CβG^+^OVA-immunized mice displayed stronger antigen-specific OT-I T cell proliferation and activation than those from mice immunized with OVA alone. Comparable responses were detected using the three adjuvants (Figure S5). We concluded that CβG is able to enhance antigen-specific CD8^+^ T cell responses *in vivo*.

To study CβG capacity to induce local inflammation, mice were immunized either with MPL, LPS, CβG or Poly I/C by skin intradermal injection. At 48 h post-treatment, both the adjuvant-treated and untreated ears were collected for histological analysis of cutaneous inflammation (Figure S6). The skin of adjuvant-treated mice revealed marked increase in ear thickness accompanied by inflammatory cell infiltration (Figure S6). The inflammation was observed in all mice immunized with different adjuvants. Animals developed acute diffuse dermatitis characterized by heavy neutrophilic infiltration associated with hyperemia of dermal capillaries with neutrophilic margination and well-developed edema of the dermis (Figure S6). Similarly to other known adjuvants, CβG is capable of inducing local inflammation.

### *Brucella* CβG activates human DC subsets

**Figure 4:**
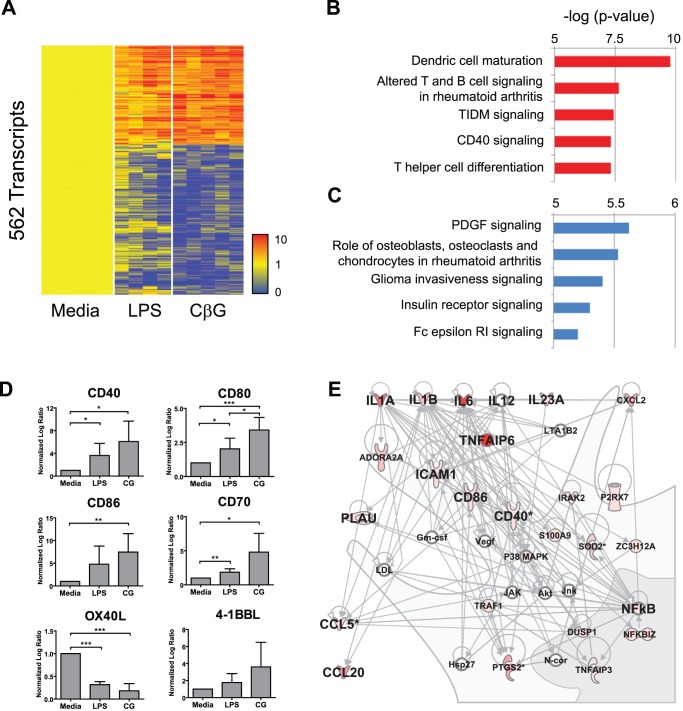
*B. melitensis* CβG induces over-expression of mRNA transcripts linked to DC maturation and T cell activation in human blood mDC. (**A**) Transcripts significantly over/under expressed 2-fold in mDC activated 6 h *in vitro* with CβG (5 donors, data normalized to 6 h activation with media control per donor). (**B**) Top canonical pathways identified by Ingenuity Pathway Analysis (IPA) for genes over-expressed in CβG-activated mDC. (**C**) Top canonical pathways identified by Ingenuity Pathway Analysis (IPA) for genes under-expressed in CβG-activated mDC. (**D**) Bar charts representing the mean normalized log ratio expression of T cell costimulatory molecules mRNA in mDC activated for 6 h with media (n = 5), LPS (n = 4) or CβG (CG) (n = 5). Bars represent the standard deviation. (**E**) Top transcriptional network identified by IPA for genes over-expressed in CβG-activated mDC. Increase in color intensity represents increase in fold-change compared to 6 h media control treatment. ***p<0.001, **p<0.01, *p<0.05.

**Figure 5:**
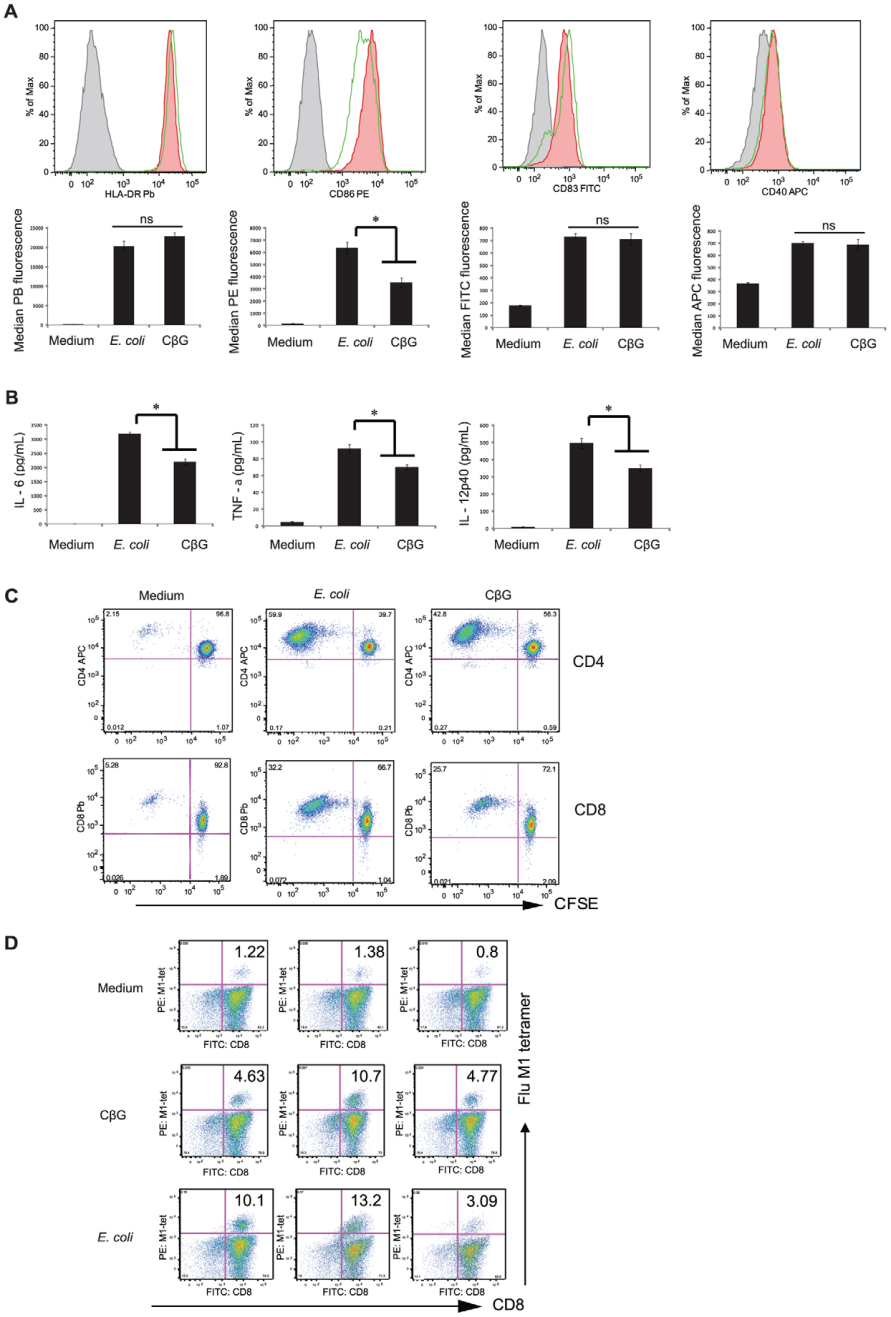
*B. melitensis* CβG-treated human blood mDC enhance CD4^+^ and CD8^+^ T cell responses. mDC were stimulated overnight with cell culture medium, 0.25 µM of *E. coli* LPS or 0.25 µM of *B. melitensis* CβG. Surface expression of HLA-DR, CD83, CD40 and CD86 was quantified by flow cytometry (**A**). Cytokine levels in culture supernatants were measured by Luminex (**B**). Experiments were performed on 4 different donors. The data for one representative are shown. * p<0.05, ns: not significant. (**C**) CFSE-labeled allogeneic naïve CD4^+^ T and CD8+ T cells were co-cultured with mDC for 7 days. Cell division was assessed by CFSE-dilution assay. Experiments were performed on 4 different donors. The data for one representative in triplicates are shown. (**D**) Autologous CD8^+^ T cells were co-cultured with mDC loaded with heat-inactivated influenza virus (PR8) for 7 days. Cells were stained with anti-CD8 antibody and Flu-M1 tetramer. Experiments were performed on 4 different donors. The data for one representative in triplicates are shown.

We next analysed whether CβG was able to activate different human DC subsets (Table 1). We included myeloid DC isolated from blood, dermis and epidermis [28], [29] as well as DC generated in vitro by culturing monocytes in the presence of GM-CSF with either IL-4 or IFNα. In response to CβG, all tested DC showed increased expression of HLA-DR, CD40, CD86, and CD83 and increased secretion of IL-12, IL-6 and TNF-α (Figures 4 and 5). Notably, CβG did not activate pDC as measured by cell surface phenotype and secretion of IFN and pro-inflammatory cytokines such as TNF-α and IL-12p70.

**Table 1:** Different human DCs subsets activation by *B. melitensis CβG*.

DC subset	DC activation
Blood mDC	+++
Blood pDC	−
IL-4 DC	++
IFNα DC	++
Skin CD1a+ DC	+++
Skin CD14+ DC	+++

*B. melitensis CβG* activation effect on different subsets of human DC was determined. The phenotype of cell activation was analysed by the up-regulation of classical activation markers at the cell surface (HLA-DR, CD40, CD86, and CD83) and the secretion of pro-inflammatory cytokines (IL-12, IL-6 and TNF-α).

To eventually distinguish the effects of LPS from those of CβG, blood mDC from five donors were activated for 6 h with either LPS or CβG and the early transcriptional responses was assessed using microarray profiling. 562 transcripts were significantly modulated in CβG-treated mDC as compared to media controls (Figure 4A). These genes displayed a similar expression profile in LPS-treated mDC, albeit with lesser global intensity as measured by the molecular distance to media. Statistical comparison (Ttest, p-value 0.01, no correction) between CβG-treated and LPS-treated mDC yielded 133 differentially regulated transcripts (data not shown), highlighting the similarities and the differences between the two stimuli. Ingenuity Pathway Analysis (IPA) identified DC maturation as the most significantly represented canonical pathway among genes over-expressed in CβG-treated mDC (Figure 4B), and PDFG signalling as the most represented pathway among genes under-expressed in CβG-treated mDC (Figure 4C). Furthermore, CβG-treated mDC displayed increased transcription of the co-stimulatory molecules CD40, CD80, CD86, CD70 and 4-1BBL, but decreased transcription of the Th2 co-stimulatory molecule OX40-L (Figure 4D). The most significantly over-expressed transcripts in CβG-treated mDC are represented as a network (Figure 4E) depicting a strong pro-inflammatory response network. Overall, the global transcriptional changes that CβG elicit in mDC support the concept that this molecule might enhance DC-dependent T cell responses.

### CβG activated blood mDC elicit CD8^+^ T cell priming

We focused our attention to the effects of CβG on human blood mDC. Like *E. coli* LPS, CβG increased the surface expression of CD40, CD83, CD86 and MHC II (Figure 5A). Furthermore, CβG-treated mDC secreted high levels of IL-6, TNF-α and IL-12 (p40) (Figure 5B) and were efficient at inducing allogeneic naïve CD4^+^ and CD8^+^ T cell proliferation (Figure 5C). *E. coli* LPS-activated DC were slightly more potent than CβG-activated DC at inducing the proliferation of naïve allogeneic T cells (Figure 5C).

DC loaded with heat inactivated influenza virus can cross-present the Flu MP antigen to CD8+T cells as 0.8–1.38% of cocultured CD8^+^ T cells are specific for Flu-MP as assessed using peptide-MHC Class I tetramers. Activation of the mDC with both CβG and *E. coli* LPS resulted in a considerably increased MP-specific CD8^+^ T cell response (Figure 5D). This indicates that CβG can enhance secondary CD8^+^T cell responses.

**Figure 6:**
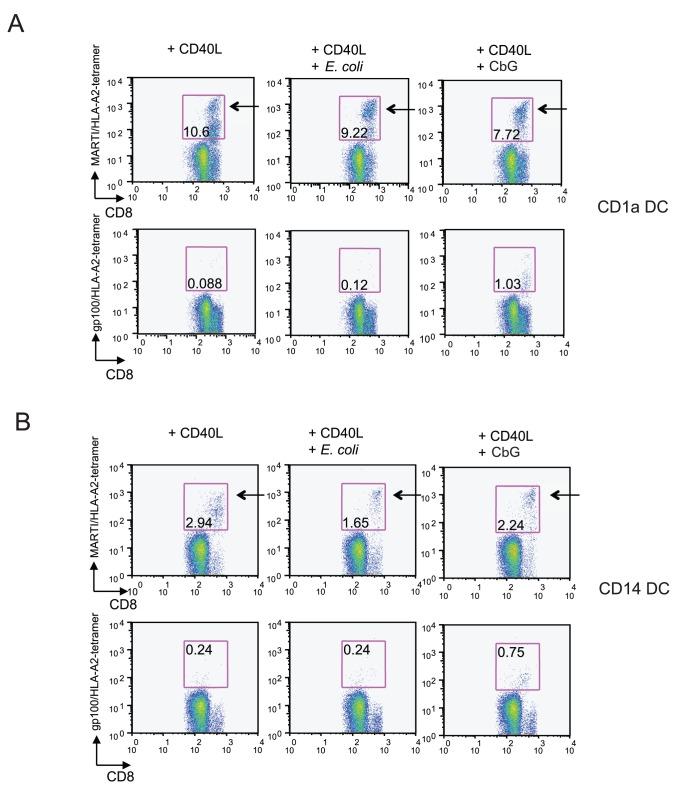
*B. melitensis* CβG-treated human skin DC enhance gp100-specific naïve CD8^+^T cell priming. (**A**) CD1a^+^ skin DC were stimulated overnight with cell culture medium, 0.25 µM of *E. coli* LPS or 0.25 µM of *B. melitensis* CβG and loaded with either MART-1 26–35 (27 L) peptides or gp100 peptide. Skin DC were then co-cultured wih autologous CD8^+^ T cells for 10 days in the presence of IL-2 and IL-7. Cells were stained with anti-CD8 antibody and tetramers. Experiments were performed on 4 different donors. The data for one representative in triplicates are shown. (**B**) CD14^+^ skin DC activated by either medium or *E. coli* LPS or *B. melitensis* CβG and loaded with either MART-1 26–35 (27 L) peptides or gp100 peptide. Autologous CD8^+^ T cells were co-cultured with these DC for 10 days in the presence of IL-2 and IL-7. Cells were stained with anti-CD8 antibody and tetramers. Experiments were performed on 4 different donors. The data for one representative in triplicates are shown.

To assess whether CβG can enhance the priming of naïve CD8^+^ T cells by DC, we chose the melanoma-derived antigens MART-1 and gp100. The experiments illustrated in Figures 6A and 6B have respectively been performed with human skin CD1a^+^ and CD14^+^ DC [28]. Priming of naïve CD8^+^T cells requires activation of the DC through CD40 and addition of IL-2 and IL-7 to the cultures. Further activation of DC with LPS or CβG did not enhance the response to the relatively abundant MART-1 T cells. However, CβG enhanced the priming to the less frequent gp100-specific naïve T cells (Figure 6).

**Figure 7:**
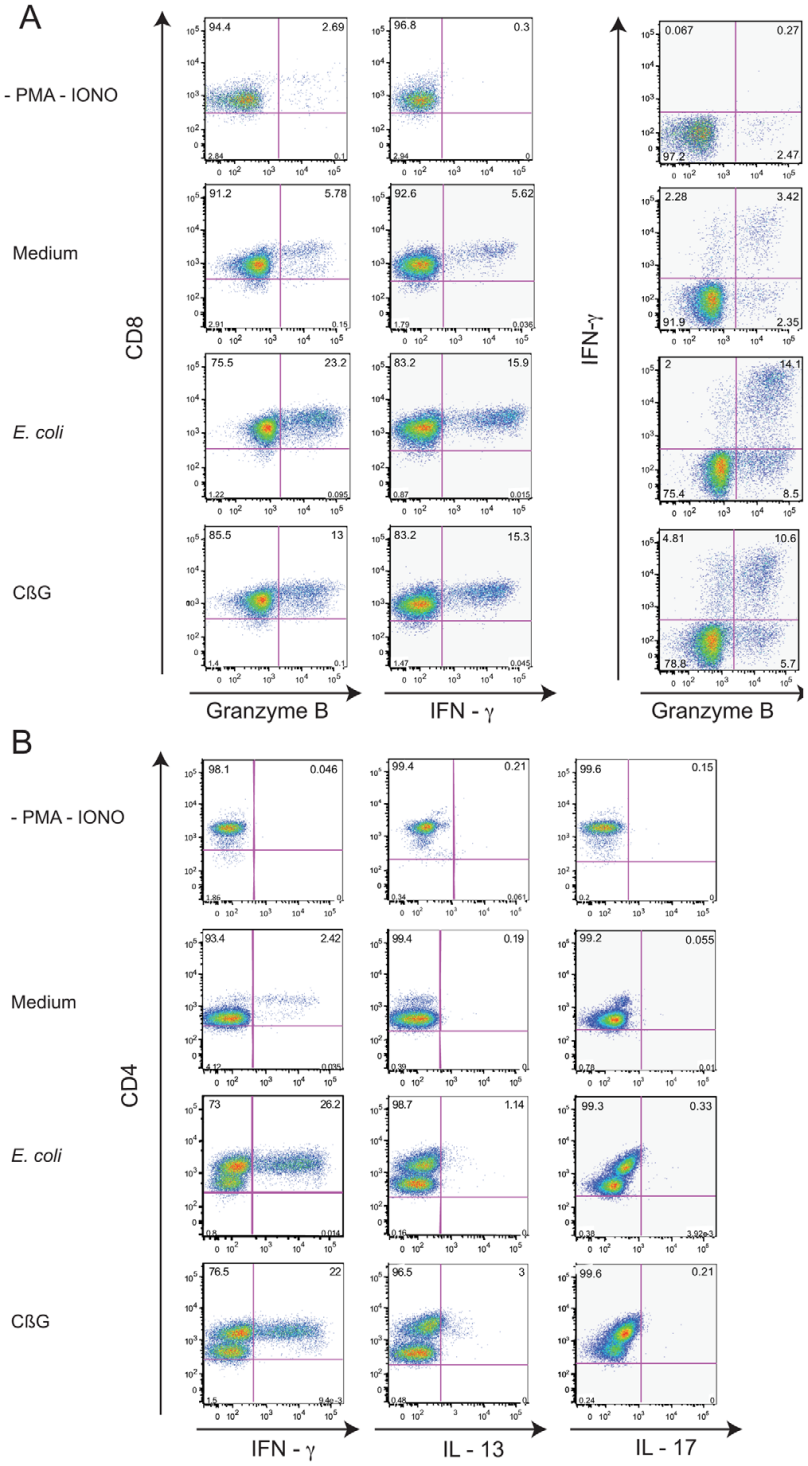
*B. melitensis* CβG induces the synthesis of effector molecules in human T cells. Human blood mDC were co-cultured with allogeneic naïve CD4^+^ T and CD8^+^ T cells. After 7 days, cells were incubated for 6 h with PMA/Ionomycin in the presence of brefeldin A. The intracellular levels of IFN-γ and granzyme B in CD8^+^ T cells (**A**) and IFN-γ, IL-13 and IL-17 in CD4^+^ T (**B**) and were analysed by flow cytometry. Experiments were performed on 4 different donors. The data for one representative are shown.

Naïve CD8^+^ T cells were also cultured for seven days with allogeneic mDC that were activated or not with either LPS or CβG (Figure 7A). CβG-activated mDC were also capable of inducing naïve CD8^+^ T cells to express high levels of IFNγ and granzyme B in CD8^+^ T cells when compared to unactivated mDC (Figure 7A).

Thus, CβG-activated mDC are able to activate CD8^+^ T cells.

### *Brucella* CβG increases CD4^+^ T memory responses in PBMC from HCV cured and TB patients

To characterize the CD4^+^ T cells exposed to CβG activated mDC, CD4^+^ T cells were cultured with allogeneic blood mDC activated or not with either LPS or CβG for seven days (Figure 7B). The cultured T cells were then restimulated with PMA/Ionomycin and stained for intracellular IFN-γ, IL-13, and IL-17. Both LPS and *B. melitensis* CβG-activated mDC polarized CD4^+^ T cells into IFN-γ-expressing Th1 cells (Figure 7B). In addition, both *E. coli* LPS and CβG-activated DC induced a minor sub-population of naïve CD4^+^ T cells to differentiate into IL-13^+^ CD4^+^ T cells. Naïve CD4^+^ T cells co-cultured with either *E. coli* LPS-activated or CβG-activated mDC did not express IL-17 (Figure 7B). Thus, CβG-activated mDC induce Th1 responses.

**Figure 8:**
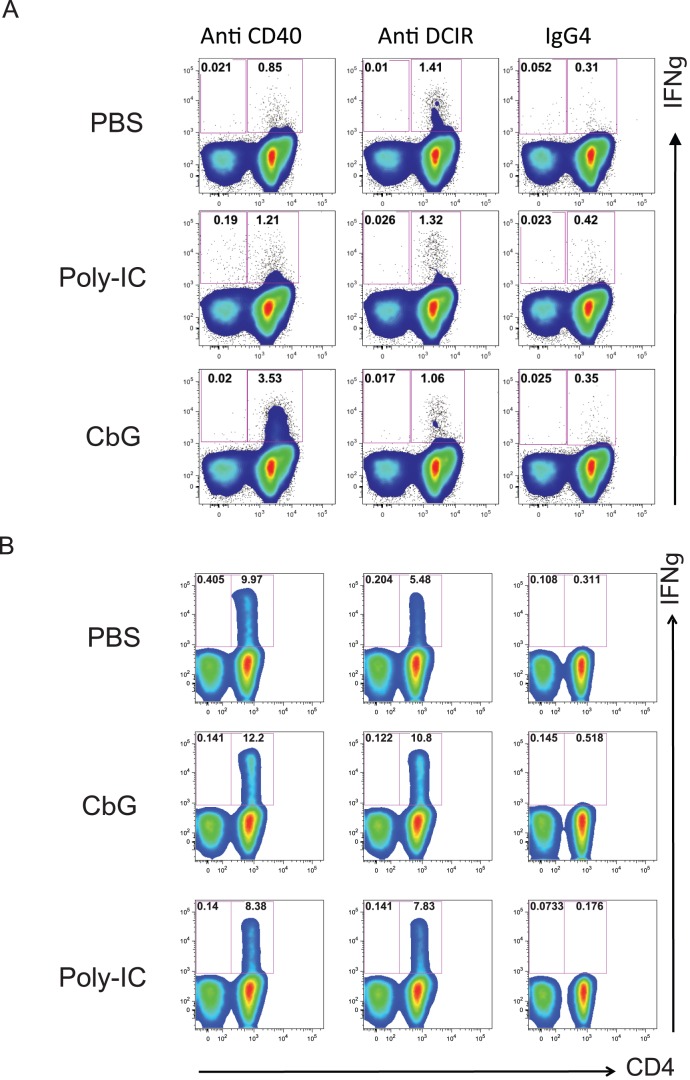
*B. melitensis* CβG potentiates CD4^+^ T memory responses in HCV and TB patients. (**A**) CβG induces CD3^+^CD4^+^IFNγ^+^ T cells after anti-CD40 HCV-NS3HelB DC-targeting of PBMC in HCV cured patients. HCV antigens from NS3 Helicase HelB construct were delivered to IFNα DC through anti-CD40 or DCIR-Ig4 humanized recombinant antibodies. IFNα DC were targeted with anti-CD40-NS3HelB (5 nM), anti-DCIR-NS3HelB (5 nM) or Ig4 control antibody (5 nM) in the presence of Poly I:C (25 µg/ml) or CβG (10 µg/ml) before co-culture for 10 days with PBMC from chronic HCV-infected patients cured after IFNα-Ribavarin therapy. Cells were stimulated for 6 h with peptide clusters (10 µM; 10 peptides of 15-mers) covering HCV NS3 HelB constructs. PBMC were stained for measuring the frequency of peptide-specific CD3^+^CD4^+^IFNγ^+^ T cells and analyzed by flow cytometry. (**B**) CβG increases CD4^+^T cell memory responses induced by anti-CD40 or anti-DCIR coupled to Ag85BD41-ESAT6-Rv1980D24 antigens in PBMC from TB patients. IFNα DC were loaded on with IgG_4_ humanized recombinant mAb Ag85BD41-ESAT6-Rv1980D24 (1 pM) either in the presence of medium versus Poly I:C (2.5 µg/ml) or CβG (1 µg/ml). After 10 days of co-culture with PBMC from acute TB patients, cells were re-stimulated with peptides at 2.5 µM covering the entire ESAT6 protein (22 peptides of 15–16 mers) for 6 h and the magnitude of the immune recall response in terms of the percentage of CD3^+^CD4^+^IFNγ^+^ T cells was analyzed by flow cytometry.

DC targeting approach consists in delivering antigens directly to DC *in vivo* using chimeric proteins composed of an anti-DC receptor antibody coupled to a selected antigen [30]. The selection of the appropriate adjuvant is a critical parameter for the induction of the desired type of immune response. PBMC from cured chronic HCV infected patients were cultured for ten days with monocyte-derived DC and recombinant humanized anti-CD40 or anti-DCIR fused to the HCVNS3 antigen with or without CβG or Poly-IC. Antigen-specific responses were measured by exposing the cultured cells to specific peptides clusters and stained for intracytoplasmic IFNγ expression. Low IFN-γ levels were observed in PBMC cultured with DC and control IgG4 with or without the adjuvants (Figure 8A). Moreover DC targeting by anti-CD40 and anti-DCIR enhanced IFN-γ production by CD4^+^ T cells. Potent memory CD4^+^ T (Figure 8A), but not CD8^+^ T cell (not shown) responses were observed after DC-PBMC co-culture in these conditions.

Few CD4^+^ T cell responses could be induced when anti-CD40 targeted-DC were treated with Poly I:C, as demonstrated by the presence of some CD3^+^CD4^+^IFN-γ^+^ T cell (1.21% of parents cells, Figure 8A). However, CβG-treated DC using anti-CD40 targeting induced a dramatic increase of CD3^+^CD4^+^INF-γ^+^ cells (3.53% of parent cells compared to 0.85% without stimulation, Figure 8A). When anti-DCIR vaccine targeting was used, we could not observe any difference in CD4^+^INF-γ^+^ T cell population with or without stimulation (Figure 8A).

We next studied CβG effect in DC targeting experiments with PBMC from acute TB patients. For this, we used humanized anti-CD40 or anti-DCIR antibodies coupled to Ag85BD41-ESAT6-Rv1980D24 *Mycobacterium tuberculosis* antigens developed by the ANRS (French agency of research against AIDS and viral hepatitis). DC targeting with control IgG4 and the specific peptides in the presence of adjuvants triggered IFN-γ production by CD4^+^ T cells (Figure 8B). This induction was increased in the presence of CβG upon anti-CD40 and anti-DCIR targeting. The stimulation by Poly I:C slightly enhanced IFN-γ production only upon anti-DCIR targeting (Figure 8B).

Taken together, these data show that *Brucella* CβG increases CD4^+^ T memory responses after DC targeting of PBMC in HCV cured and acute TB patients.

## Discussion

Vaccination represents the most effective strategy to combat infectious diseases. Vaccines are composed of antigens and adjuvants, which activate antigen-presenting cells which then triggers the activation, differentiation and expansion of antigen-specific T and B lymphocytes [15], [31]–[33]. The number of approved adjuvants effective in humans is very limited since they are mostly based on alum and on emulsions. Ideally, adjuvants should elicit a selected immune response (i.e. cellular or humoral immunity depending on the requirements for protection), be safe (sufficiently immunogenic, without excessive inflammation) and cost-effective [16], [18], [31]. The benefits of adjuvant incorporation into any vaccine formulation should significantly outweigh the risks of adverse reactions. Unfortunately, potent adjuvant action is often correlated with increased toxicity, as exemplified by Freund's complete adjuvant or LPS. Thus, one of the major challenges in human adjuvant development is to identify compounds that enhance vaccine antigen induced responses with maximum tolerability and safety [31]. In particular, there is a high demand for adjuvants that stimulate cellular immunity [15], [16], [18]. Here, we demonstrate that *Brucella* CβG is a non-immunogenic and non-toxic molecule capable of triggering the activation of cellular responses *in vivo*. Moreover, *Brucella* CβG dramatically increases specific memory CD4^+^ T cell responses of human PBMC induced by DC-targeting fusion proteins expressing either HCV antigens or *Mycobacterium tuberculosis* antigens. Based on these data, we propose that *Brucella* CβG is a candidate adjuvant that might be used in humans.

There has been an interest to identify TLR4 agonists with a dampened toxicity. A recent example of lipid A analog is MPLA known to activate immune cells with similar properties of the LPS but less toxic and non immunogenic [34], [35]. Our results show that CβG-induced DC maturation is dependent on TLR4 as well as Myd88 and TRIF adaptor molecules. While CβG activates all human mDC subsets, it does not activate pDC, which is consistent with their lack of TLR4 [24]–[26]. However, what differentiates CβG from MPLA and Ploy I:C is that CβG induces an early immune response (Figure 3). This might have a significant impact on the quality of the immune response to vaccines [36].

We previously showed that *Brucella* CβG is capable of interacting with lipid rafts and modulate their organization [6]. Lipid rafts are plasma membrane microdomains enriched in cholesterol and sphingomyelin that are involved in intracellular signalling and membrane transport. In particular, lipid rafts are involved in the regulation and activation of several important immune receptor complexes such as the TLR4 complex [37]. [38]. It is thus possible that *Brucella* CβG triggers TLR4-dependent signalling through its effect on cholesterol in lipid rafts as it has been suggested for alum [15]. Indeed, alum recognition may occur indirectly through the release of endogenous uric acid. Recently, it was shown that monosodium urate crystals activate DC by interacting with the cell membrane, which leads to plasma membrane lipid sorting probably via interaction with cholesterol [15].

Herein, we observed that in contrast to *E. coli* LPS [27], *Brucella* CβG-induced BMDC maturation was independent of the GPI-anchored protein CD14. Actually, the synthetic lipid A compound CRX-527 does not require CD14 to engage MyD88-dependent and TRIF/IRF3-dependent pathways downstream TLR4 [39]. Furthermore, the uropathogenic *E. coli* (UPEC) triggers innate responses during urinary tract infection in a TLR4-dependent and CD14-independent manner both in mice and humans [40]. These results clearly indicate that CD14 is not required for TLR4-dependent cell activation. The detailed signalling pathway involved in CβG-dependent cell activation will require further studies including the discovery of putative co-receptors associated to TLR4. Until now, we know that neither dectin-1 nor TLR2 contribute to the activation. Additional studies are planned to determine whether the CD14-independent CβG-dependent DC activation will have any therapeutic value.

CβG displays interesting properties such as water solubility, limited toxicity and lack of immunogenicity together with a potent DC activation capacity that can trigger CD4^+^ and CD8^+^ T cell responses. Therefore, CβG might constitute a new class of adjuvants for future vaccines.

## Materials and Methods

### Ethics

Animal experimentation was conducted in strict accordance with good animal practice as defined by the French animal welfare bodies (Law 87–848 dated 19 October 1987 modified by Decree 2001-464 and Decree 2001-131 relative to European Convention, EEC Directive 86/609). INSERM guidelines have been followed regarding animal experimentation (authorization No. 02875 for mouse experimentation). All animal work was approved by the Direction Départmentale des Services Vétérinaires des Bouches du Rhônes (authorization number 13.118). For animal exerimentation in Costa Rica, animals were handled and sacrificed according to the approval and guidelines established by the “Comité Institucional para el Cuido y Uso de los Animales” of the Universidad de Costa Rica, and in agreement with the corresponding law “Ley de Bienestar de los Animales No 7451” of Costa Rica (http://www.micit.go.cr/index.php/docman/doc_details/101-ley-no-7451-leyde-bienestar-de-los-animales.html). The animal handling and procedures were in accordance with the current European legislation (directive 86/609/EEC) supervised by the Animal Welfare Committee of the institution (protocol number R102/2007).

Patients were recruited at the Baylor Hospital Liver transplant Clinic (BHLTC, Dallas, TX) after obtaining informed consent. The study was approved by the Institutional Review Board of the Baylor Health Care System (Dallas, TX).

### Reagents

Antibodies used for immunofluorescence labelling included rabbit Rivoli antibody against murine I-A [41]. CpG (Invivogen), Pam2CSK4 (Invivogen) and curdlan (Megazyme) were used to activate DC. Antibodies used for flow cytometry included APC-CD11c, FITC-CD40, FITC-CD80, PE-CD86, PE-IA-IE (MHC class II) (Pharmingen), as well as PB-CD8, A700-CD45.2, APC-CD44, PE-Cy7-CD25, APC-CD62L (BD Biosciences and eBiosciences). The Aqua Dead Cell Stain (Invitrogen) was used to eliminate dead cells. Human mDC were sorted from PBMC of blood from healthy donors using lineage cocktail-FITC (BD Biosciences), CD123-PE (BD Biosciences), CD11c-APC (Biolegend), HLA-DR-Quantum Red (Sigma). Human mDC were stained with CD86-PE, CD83-FITC, CD40-APC and HLA-DR-PB (eBiosciences or Biolegends). 7-AAD was used to exclude dead cells. For intracellular labelling IL13-APC, INF-γ-PE-Cy7, IL-17-PE and Granzyme B-APC antibodies were used. Isotype matched controls were used appropriately. At least 100.000 events were collected on flow cytometry Canto II (BDBiosciences) or FACSAria (BDBiosciences). Flow cytometry analysis was performed using the FlowJo software. Purified cyclic glucans were obtained from *B. melitensis* 16 M or *Brucella abortus* 2308 [42] and from *Ralstonia solanacearum* (gift from Dr. J.-P. Bohin, CNRS UMR8576, Lille, France. Cells were stimulated with 100 ng/ml of *E. coli* LPS and 10 µg/ml of *B. melitensis* CβG to have the equivalent molarity of reagents (0.25 µM).

### Mice and cells

CD-1 and C57Bl/6 Ly5.1 mice from Jackson Laboratory and OT-I TCR transgenic Ly5.2 mice on C57Bl/6 background were used. C57BL/6, TLR4^−/−^, TLR2^−/−^, MyD88^−/−^, TRIF^−/−^, MyD88/TRIF^−/−^ mice were maintained at CIML animal house, France. CD14^−/−^ mice were obtained from CDTA, Orleans, France. All mice were from a C57BL/6 genetic background. Mouse bone marrow-derived DC (BMDC) and macrophages (BMDM) were prepared from 7–8 week-old female C57BL/6 mice as previously described [43].

### Human DC

Human monocyte-derived DC were generated from Ficoll-separated PBMC from healthy volunteers [44]. Monocytes were enriched from the leukopheresis according to cellular density and size by elutriation as per manufacturer's recommendations. For DC generation, monocytes were resuspended in serum-free Cellgro DC culture supplemented with 100 ng/ml GM-CSF and 500 UI/ml IFN-α. mDC (HLA-DR^+^CD11c^+^CD123^−^Lin^−^) and pDC (HLA-DR+, CD11c−, CD123+, Lin−) were sorted from fresh PBMC using FACSAria cytometer (BD Biosciences). Naïve CD4^+^ and CD8^+^ T cells (CD45RA^+^CD45RO^−^CCR7^+^) (purity>99.2%) were purified by flow cytometry sorting.

### *Brucella CβG* extraction, purification and characterization

CβG were obtained from *B. melitensis* 16 M or *B. abortus* 2308 grown and inactivated as described before [42]. For CβG extraction and purification, the protocol described before [6] was used. Briefly, a CβG-rich crude fraction was first obtained by ethanol precipitation of a hot water extract of killed bacteria, and freed from nucleic acids or proteins by digestion with DNase and RNase proteinase K. To remove LPS, the fluid was extracted with a volume of phenol (in contrast to most LPS, *Brucella* LPS and lipid A partition into the phenol phase [42] at 70°C for 30 min, the mixture chilled and centrifuged (8000× *g*, 0°C, 15 min), and the aqueous phase collected and re-extracted again with phenol under the same conditions. The new aqueous phase was dialyzed, clarified by brief centrifugation and freeze-dried. The identity of CβG was demonstrated by ^13^C-NMR spectroscopy and high-performance TLC, and the absence of *Brucella* LPS or other contaminants was demonstrated by UV-spectrophotometry, SDS-PAGE, gel immunoprecipitation, and 3-deoxy-d-manno-2-octulosonic acid analyses [42]. To further demonstrate the absence of lipid A, purified CβG were analyzed by MALDI-TOF mass spectrometry as described before [45]. Briefly, 5 mg of lyophilized *Brucella*'s CβG were resuspended in 100 µl of chloroform-methanol-water (3∶1.5∶0.25 [vol/vol/vol]) and 1 µl aliquot of was deposited on the target and covered with the same amount of 2,5-dihydroxybenzoic acid matrix (Sigma) dissolved in chloroform-methanol-water (3∶1.5∶0.25 [vol/vol/vol]). Different ratios between the samples and dihydroxybenzoic acid were used. Alternatively, matrix solution was prepared by dissolving 1 mg of 2,5-dihydroxybenzoic acid with 0.1 ml of CH_3_CN/H_2_O (3∶2, vol/vol). Analyses were performed in reflector modes and in both the positive and the negative ion modes on a Bruker Autoflex II MALDI-TOF mass spectrometer (Bruker Daltonics, Inc.). A peptide calibration standard (Bruker Daltonics) was used to calibrate the MALDI-TOF. Spectra were recorded between 1900 and 3900 Da. Each spectrum was an average of 500 shots. Mark the absence of the ion species at *m/z* 2073, 2145, 2173 that are characteristic of *Brucella* lipid A [46].

### Cyclic glucan toxicity and immunogenicity assays

LAL (Limulus ameobocyte lysate) test was used for the detection and quantification of bacterial endotoxins. Briefly, the samples were incubated with the circulating blood of horseshoe crab (the LAL) and a synthetic color producing substrate to detect endotoxins. LAL contains enzymes that are activated in a series of reactions in the presence of endotoxins. This assay is quantitative and the color intensity developed upon addition of the sample to the LAL is proportional to the amount of endotoxin in the sample and can be calculate from a standard curve. Immunogenicity of purified CβG was tested in mice and rabbits following described protocols [47]. LD50 (Lethal dose 50%) was measured in CD-1 mice injected with appropriate amounts of reagents. Animal death was recorded at 12 h, 24 h, 48 h, and 72 h post-injection. To determine the immunogenicity of *B. melitensis* CβG, Balb/C mice were immunized by either PBS or *E. coli* LPS, (10 µg/mouse) or *B. melitensis* LPS (10 µg/mouse) or *B. melitensis* CβG (10 µg/mouse). The primary antibody response was measured 21 days after immunization. Mice were boosted 45 days with the same molecules (5 µg/mouse) to measure the secondary antibody response. Antibody responses were determined by an indirect ELISA.

### mRNA extraction and hybridization

After culture, human mDC were lysed in RLT buffer and stored at −80°C until further processing. Total RNA was extracted using the mirVana miRNA Isolation Kit, from Ambion. Following RNA extraction, RNA concentration was measured using a Nanodrop 1000 (Nanodrop Technologies, Wilmington, DE) and the RIN was measured with an Agilent 2100 Bioanalyzer (Agilent, Palo Alto, CA) for quality control purposes. All samples with RIN values greater than seven were retained for further processing. 250 ng of total RNA were amplified and labelled with the Illumina TotalPrep-96 RNA amplification kit (Ambion, Austin, TX). 750 ng of amplified labelled RNA were hybridized overnight to Illumina HT12 v4 Beadchip arrays (Illumina, San Diego, CA). Following hybridisation, each chip was washed, blocked, stained and scanned on an Illumina iScan following the manufacturer's protocol. The dataset described in this manuscript is deposited in the NCBI Gene Expression Omnibus (GEO, http://www.ncbi.nlm.nih.gov/geo, GEO Series accession number GSE32023).

### Microarray analysis

Transcripts present in at least one of the samples as defined by significant chip detection value were used as a starting list. Non-parametric test was applied between CβG-treated mDC and medium-treated mDC from 5 donors, false discovery rate: 0.01, with Benjamini-Hochberg correction. Pathway analysis was conducted with Ingenuity Pathway Analysis (IPA) software, Ingenuity Systems, Inc, Redwood City, CA. The molecular distance to medium (MDTM) quantifies the global transcriptional perturbation of a group of transcripts in a specific sample as compared to its reference control(s). It is calculated as follows. For an arbitrary list of transcripts normalized to their reference control(s), the absolute fold changes greater or equal to 2 for a specific sample are summed up.

### Cytokine measurement

Murine IL-12 and TNF-α were quantified in culture supernatants of stimulated DC by sandwich enzyme-linked immunosorbent assays (ELISA) according to the manufacturer's protocol (Abcys). Human cytokine (IL-6, TNF-α, and IL-12p40) were determined using the BeadLyte cytokine assay kit (Upstate, MA).

### Cytometric Bead Array (CBA) assay

Mice were divided into groups each of 5 mice and were immunized i.p. with either: 50 µl of PBS, 20 µg of Monophosphoryl Lipid A (MPLA) (InvivoGen) in 50 µl of PBS, 200 µg of CβG in 50 µl of PBS, 10 µg of LPS in 50 µl of PBS, 50 µg of Poly I/C in 50 µl of PBS. For *in vivo* cytokine measurements, mice were bled submandibularly at 6 h, 24 h and 72 h after immunization and sera collected. Supernatants were then harvested at each time point and kept at −20°C. To determine the amounts of cytokines produced in the sera, a CBA assay (BD Biosciences) that detects IL-6, IL-10, MCP-1, IFN-γ, TNF-α and IL-12p70 in a single sample was used. The sera from immunized mice were incubated with a mixture of specific capture antibodies that were coupled to the beads containing specific amounts of PE fluorescence intensity. The 6 different fluorescence intensities of PE were detected by flow cytometry and cytokine concentration in the samples was quantified from a standard curve according to manufacturer's protocol. 10.000 events were analyzed by flow cytometry Canto II (BDBiosciences) and the data were analyzed using the FlowJo software.

### Human CD4^+^ and CD8^+^ T cell responses

Blood mDC were co-cultured with CFSE-labeled allogeneic naïve CD4^+^ T and CD8^+^ T cells. The expression of intracellular cytokines and granzyme B were measured after 6 h of cell stimulation by PMA and Ionomycine, in the presence of Brefeldin A. Blood mDC from HLA-A0201^+^ healthy donors were loaded with a multiplicity of infection (MOI) of 0.2 of heat-inactivated influenza virus (PR8) for 2 h at 37°C. Autologous CD8^+^ T cells were mixed and cultured for 7 days in the presence of 20 units/ml IL-2. Cells were then stained with anti-CD8 antibody and tetramer (HLA-A*0201-Flu M1_58–66_). MART-1-specific CD8^+^ T cell responses were measured after co-culturing with CD1a^+^ and CD14^+^ skin DC loaded with 10 µM 15-mer MART-1 peptide- containing the immunodominant epitope MART-1_26–35 (27L)_ or with gp100 peptide epitope for 10 days. Skin DC were purified as previously described [28]. To activate individual DC subsets we used 0.25 mM of either *E. coli* LPS or CβG.

Autologous IFNα DC loaded with vaccine candidates (for Mycobacterium: ESAT6 protein, for HCV: antigens from NS3 Helicase helB construct) were co-cultured with PBMC from HCV or TB patients and incubated for 10 days. T-cell specific responses elicited by vaccine candidate loaded-DC were assessed by restimulating PBMC with peptide clusters

### Adoptive transfer of OT-I T cells and immunization

OT-I transgenic cells that express TCR specific for an H-2K^b^ restricted CD8^+^ T cell epitope from OVA were used. Lymph nodes from OT-I Ly5.1 mice were harvested and digested with collagenase type I (Sigma) at 37°C for 30 min. CD8^+^ T cells were then negatively sorted by using mouse CD8 negative isolation kit (Dynal). CD8^+^ T cells were labeled with 10 µM CFSE (Invitrogen) and transferred intravenously (i.v.) into naive congenic C57Bl/6 Ly5.2 recipient mice. At 24 h, recipient mice were immunized subcutaneously (s.c.) either with 30 µg OVA (EndoGrade) alone in endotoxin free PBS or 30 µg OVA mixed with 200 µg of CβG or 30 µg OVA mixed with 50 µg poly I:C (Sigma) or 30 µg OVA mixed with 20 µg MPLA (InvivoGen).

### Histology

For histological assessment of cutaneous inflammation, intradermal injection of mouse ear skin was performed. C56BL/6 mice were immunized in the right ear with either: 20 µg of Monophosphoryl Lipid A (MPL) in 10 µl of PBS, 200 µg of CβG in 10 µl of PBS, 10 µg of LPS in 10 µl of PBS, 50 µg of Poly I/C in 10 µl of PBS. PBS was injected in the left ear as negative control. The animals were sacrificed 48 h later and both the adjuvant-treated and untreated ears were collected for further determination. Ears were fixed in 10% neutral buffered formalin for 48 h, embedded in paraffin, then sectioned at 4 µm at two levels separated by 1 mm interval and stained with hematoxylin and eosin. Normal skin histology was evaluated in PBS-inoculated contralateral ear for each mouse.

### Statistical analysis

All experiments were carried out at least 3 independent times and all the results correspond to the means ± standard errors. Statistical analysis was done using two-tailed unpaired Student's *t* test. Significance was defined when *P* values were <0.05.

### Supplementary material

See legends of Figures S1, S2, S3, S4, S5 and S6.

## Supporting Information

Figure S1 **Bone marrow-derived murine dendritic cells infected with** ***cgs-*** **mutant display a low maturation profile.** Mouse DC were infected for 24 h with *Salmonella thyphimurium* S12023 virulent strain, *Brucella abortus* 2308 virulent strain or isogenic *cgs- B. abortus* mutant. (**A, B**) IL-12 and TNFα levels in supernatant were measured by ELISA after infection, respectively. Data represent means ± standard errors of at least 3 independent experiments. *p<0.05 *cgs-* mutant compared to the *wild type* values, ns: not significant.
(EPS)

Figure S2 **MALDI-TOF mass spectrometry analysis of CβG preparations.** The spectra show six main signals at *m*/*z* 2,795, 2,957, 3,119, 3,268, 3,444, and 3,607 consistent with those expected for CβG molecules composed of 17–22 glucose residues, as previously described. (**A**) Positive-ion MALDI-TOF MS. (**B**) Negative-ion MALDI-TOF MS. Mark the absence of the ion species at *m/z* 2073, 2145, 2173 that are characteristic of *Brucella* lipid A.
(EPS)

Figure S3 ***B. melitensis*** **CβG induces mouse DC maturation in a dose-dependent manner.** (**A**) Mouse DC were stimulated for 8 h (white) and 24 h (black) with medium, *E. coli* LPS (0.25 µM) or *B. melitensis* CβG (0.025 µM, 0.25 µM, 2.5 µM). MHC II, CD80, CD40 and CD86 surface levels were analyzed by flow cytometry. Graphs represent median of fluorescence ± standard error of four independent experiments. (**B**) Mouse DC were treated in triplicate for 8 and 24 h with either medium, *E. coli* LPS (0.25 µM) or *B. melitensis* CβG (0.025 µM, 0.25 µM, 2.5 µM). IL-12 and TNFα levels in the supernatants were measured by ELISA. Data represent means ± standard errors of at least 3 independent experiments. *p<0.05., ns: not significant.
(EPS)

Figure S4 ***B. melitensis*** **CβG is not immunogenic in mice.** (**A**) Balb/C mice were immunized by either PBS or *E. coli* LPS, (10 µg/mouse) or *B. melitensis* LPS (*Brucella* LPS, 10 µg/mouse) or *B. melitensis* CβG (CβG, 10 µg/mouse). The primary antibody response (white bars) was measured 21 days after immunization. Mice were boosted 45 days with the same molecules (5 µg/mouse) to measure the secondary antibody response (black bars). 3 independent experiments were performed (n = 5), *p<0.05. (**B**) Levels of pro-inflammatory cytokines are strongly reduced in the sera of CβG-immunized mice. C57Bl/6 mice (n = 5) were immunized either with PBS, MPL, CβG, LPS or Poly I/C. After 6 h, 24 h and 72 h of immunization, mice were bled and the cytokine levels were measured in the sera by CBA. The levels of IL-6, IL-10, MCP-1, IFNγ, TNFα and IL-12p70 are presented. Data represent means ± standard errors from 5 samples. ***p<0.001, **p<0.01.
(EPS)

Figure S5 ***B. melitensis*** **CβG induces cellular immunity** ***in vivo*** **.** CD8^+^ Ly5.2 CFSE^+^ T cells were transferred intravenously (i.v.) into naive congenic C57Bl/6 Ly5.1 recipient mice. 24 h after OT-I adoptive transfer, recipient mice were immunized subcutaneously (s.c.) either with PBS (grey) or 30 µg OVA in PBS or 30 µg OVA mixed with 200 µg of CβG or 30 µg OVA mixed with 50 µg Poly I:C or 30 µg OVA mixed with 20 µg MPLA. At day 6 post-immunization, the OVA-specific OT-I T cell activation in the draining popliteal lymph nodes of immunized mice was investigated by analyzing the up-regulation of CD25 and the down-regulation of CD62L by flow cytometry. The median fluorescence for each marker is indicated under histograms. Endogenous CD8^+^ T cell population (in grey). Data are representative of one experiment among three different experiments.
(EPS)

Figure S6 **CβG triggers a local skin inflammation.** Mice were immunized either with PBS, MPL, CβG, LPS or Poly I/C. At 48 h post-treatment, both the adjuvant-treated and untreated ears were collected for histological analysis of cutaneous inflammation. Hematoxylin- and eosin-stained sections of adjuvant-immunized mice revealed marked increase in ear thickness accompanied by inflammatory cell infiltration.
(TIF)
