# Supplementary figures and images for: Brucella β 1,2 Cyclic Glucan Is an Activator of Human and Mouse Dendritic Cells
Source: PLoS Pathog. 2012 Nov 15;8(11):e1002983. doi: 10.1371/journal.ppat.1002983 (PMC3499565; doi:10.1371/journal.ppat.1002983)

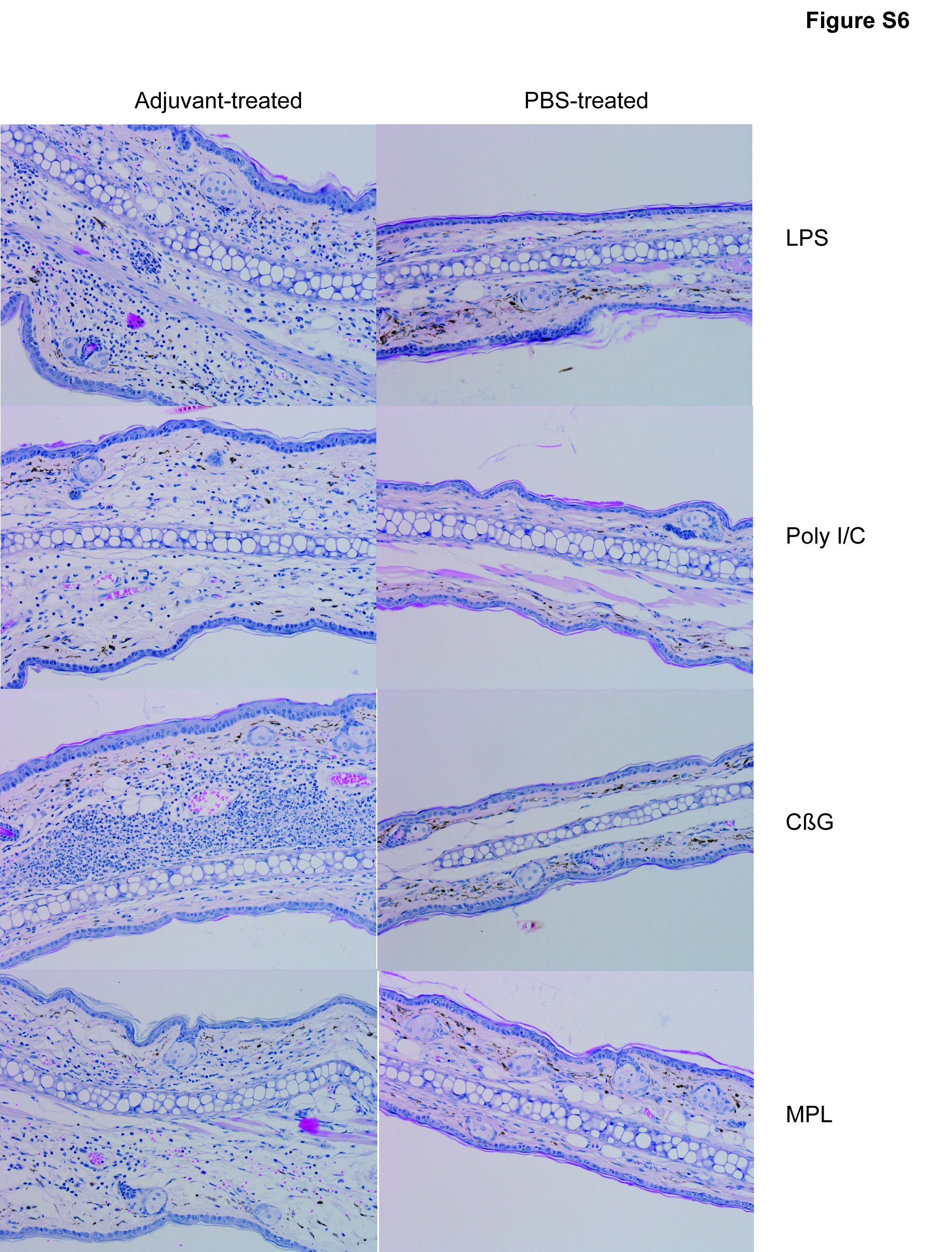

Supplement: Figure S6 — CβG triggers a local skin inflammation. Mice were immunized either with PBS, MPL, CβG, LPS or Poly I/C. At 48 h post-treatment, both the adjuvant-treated and untreated ears were collected for histological analysis of cutaneous inflammation. Hematoxylin- and eosin-stained sections of adjuvant-immunized mice revealed marked increase in ear thickness accompanied by inflammatory cell infiltration. (TIF) [file ppat.1002983.s006.tif]
